# Development of Gap-Recombinase Polymerase Amplification Combined with CRISPR-Cas12a (Gap-RPA/CRISPR-Cas12a) for Rapid Screening of α^0^-Thalassemia Southeast Asian (--^SEA^) Deletion and Thai (--^THAI^) Deletion

**DOI:** 10.3390/biology15181579

**Published:** 2026-09-08

**Authors:** Phanupong Changtor, Siriphat Muangpa, Nonglak Yimtragool

**Affiliations:** Department of Biology, Faculty of Science, Naresuan University, Phitsanulok 65000, Thailand; phanupongc@nu.ac.th (P.C.); siriphatm64@nu.ac.th (S.M.)

**Keywords:** α^0^-thalassemia, DETECTR, isothermal, lateral flow assay, point-of-care

## Abstract

The α^0^-thalassemia Southeast Asian (--^SEA^) and Thai (--^THAI^) deletions comprise a significant global health burden, with high prevalence in Southeast Asia. Conventional methods like polymerase chain reaction (PCR) are limited in resource-poor settings due to complexity, time, and cost. This assay was developed as a rapid and simple option for point-of-care (POC) applications. Three assays specific for wild-type DNA, --^SEA^ and --^THAI^ deletions utilized recombinase polymerase amplification (RPA) for rapid DNA amplification at a constant low temperature of 37 °C, with a run time of 10 min, eliminating the need for a thermal cycler. The amplified products were detected using the highly specific CRISPR-Cas12a system and read visually via fluorescence or combined with a lateral flow assay (LFA) in 30 min. Successful engineering of a synthetic PAM site enabled Cas12a recognition of the --^SEA^ target despite the absence of a suitable natural PAM. The platform demonstrated high accuracy in 74 blinded clinical samples, achieving a diagnostic accuracy of 98.20%, a sensitivity of 94.59%, and a specificity of 100% across 222 per-target evaluations. Detection from non-invasive samples achieved 80% to 100% accuracy, depending on the extraction method and readout format used. Our platform showed potential to meet several ASSURED criteria for diagnostic tests in resource-limited settings, demonstrating high potential for widespread use in genetic screening for α^0^-thalassemia (--^SEA^) and (--^THAI^).

## Simple Summary

Severe α^0^-thalassemia is a genetic blood disorder that creates a major health burden in Southeast Asia. Current testing methods are too complex, slow, and expensive for communities with limited resources. To address this problem, we developed a fast, simple, and affordable test. For the --^SEA^ deletion, primer engineering successfully introduced a synthetic PAM site, enabling Cas12a detection where a suitable natural PAM was absent. Our method works at a constant temperature in under 40 min without using expensive laboratory machines. This platform was over 98 percent accurate and worked effectively using pain-free samples instead of blood. The test shows several features that are suitable for use in resource-limited settings. This is valuable to society because it provides an easy and affordable way to screen people for α^0^-thalassemia.

## 1. Introduction

Thalassemia, an inherited blood disorder characterized by the deficient synthesis of globin chains, has now become a major global health burden, manifesting in a variety of forms, ranging from asymptomatic carrier states to severe, life-threatening anemias that necessitate chronic blood transfusions [1]. Among the different types, α^0^-thalassemia, resulting from the deletion or non-deletional mutations affecting one or more of the four α-globin genes on chromosome 16 and is highly prevalent in Southeast Asia [2,3]. Two of the most widespread and clinically significant α-thalassemia mutations in Thailand are the large deletions known as the α^0^-thalassemia Southeast Asian (--^SEA^) deletion and the Thai (--^THAI^) deletion [4]. The α^0^-thalassemia genotype, predominantly (--^SEA^) and (--^THAI^) deletions, is highly prevalent in Southern China (4.64–7.91%) and across Southeast Asia (1.86–14.40%) [5,6,7]. The high carrier frequency of these deletions places a large population at risk of severe conditions, most notably Hb Bart’s hydrops fetalis, a fatal disorder that occurs when a fetus inherits two α^0^-thalassemia alleles. Therefore, accurate and accessible genetic screening for these deletions is a critical public health priority for genetic counseling, prenatal diagnosis, and effective disease management [8].

Conventional laboratory methods for initial thalassemia screening rely on hematological indices such as mean corpuscular volume (MCV) and mean corpuscular hemoglobin (MCH) [9]. These parameters provide an effective and low-cost initial assessment, but they lack the specificity to identify the underlying genetic cause and cannot differentiate between various types of microcytic hypochromic anemias. The definitive diagnosis of α^0^-thalassemia deletions currently depends on polymerase chain reaction (PCR)-based techniques, specifically gap-PCR and real-time PCR [10]. These methods, while highly sensitive and specific for detecting genetic deletions, have significant limitations that hinder their widespread application in resource-limited settings. The protocols are complex, time-consuming, and require expensive laboratory infrastructure, including thermal cyclers and gel electrophoresis equipment [10]. The need for skilled personnel to perform and interpret the assays also limits their use in small clinics or remote areas. These constraints underscore a critical unmet need for a diagnostic tool that is rapid, simple, and affordable, suitable for point-of-care (POC) applications and decentralized screening programs.

The field of molecular diagnostics has recently shifted toward isothermal nucleic acid amplification technologies, which amplify DNA at a constant temperature and eliminate the reliance on expensive thermal cyclers. The various methods developed include the widely used Loop-Mediated Isothermal Amplification (LAMP) [11], Rolling Circle Amplification (RCA) [12], and Recombinase Polymerase Amplification (RPA) [13,14]. Among these innovations, RPA stands out for its exceptional speed and low operating temperature (35–42 °C) [13,14]. RPA is highly suitable for field-deployable applications because it can be powered by a simple heat block or even body heat [15,16]. The RPA reaction operates through a unique three-enzyme system as a recombinase enzyme, which initiates the reaction by forming a nucleoprotein filament with the primers and invading the target double-stranded DNA; a single-stranded DNA-binding protein (SSB), which prevents the reannealing of the separated DNA strands; and a strand-displacing DNA polymerase, which extends the primers to synthesize new DNA [13,14]. This elegant mechanism enables the amplification of target sequences in 10–20 min [17,18], offering a rapid and robust alternative to conventional PCR. The rapid speed and simplicity of RPA have enabled its widespread application across diverse sectors. RPA has been successfully utilized for the on-site, single-tube detection of human pathogens in clinical diagnostics, as well as for routine monitoring in food safety and agricultural health without the need for extensive laboratory infrastructure [14,17,18,19].

The diagnostic power of RPA has been further enhanced by its integration with the CRISPR-Cas (Clustered Regularly Interspaced Short Palindromic Repeats-CRISPR-associated) system [20,21,22]. The Cas12a endonuclease has become a cornerstone of molecular diagnostics due to its unique mechanism. Cas12a is guided by a guide RNA (gRNA) to a specific target DNA sequence [23]. The presence of a protospacer adjacent motif (PAM) adjacent to the target sequence is essential for Cas12a to bind and activate. Once activated, Cas12a exhibits a trans-cleavage activity, meaning it can non-specifically cleave any nearby single-stranded DNA (ssDNA) molecules. This collateral activity is the basis for its use as a diagnostic sensor [23]. The applications of CRISPR-Cas12a for diagnostics are diverse and rapidly expanding. It has been used for the sensitive detection of viral RNA in diseases like COVID-19 [24], where a CRISPR-based assay known as DETECTR (DNA Endonuclease Targeted CRISPR Trans Reporter) was developed to provide rapid results from detection samples [25]. The combination of RPA and CRISPR-Cas12a creates a synergistic platform that is rapid, sensitive, specific, and robust for identifying target DNA sequences [20,21,22]. This integrated approach bypasses the need for complex thermal cycling and gel electrophoresis, allowing for DNA amplification and post-amplification analysis with minimal scientific equipment. A single-tube assay can be configured where RPA amplifies the target DNA, and the resulting amplicons activate the Cas12a enzyme. This activation leads to the trans-cleavage of a fluorescent reporter probe [25], which can be visualized with a simple UV light or a smartphone-based fluorometer. This elegant two-step process, including rapid amplification by RPA followed by high-fidelity detection by CRISPR-Cas12a, has already been applied to create highly effective diagnostic tools. Researchers have developed integrated RPA-CRISPR assays for the detection of human genetic variants, such as G6PD variants [26], as well as tuberculosis, foodborne pathogens, and COVID-19 in low-resource settings, demonstrating their practicality and superior performance compared with traditional methods [20,21,22,24,26]. Furthermore, several isothermal amplification methods have been reported for α^0^-thalassemia deletion detection. An RPA-based assay targeting the --^SEA^ and --^THAI^ deletions reported 100% sensitivity and specificity in carrier and prenatal cohorts [27]. A LAMP-based assay for --^SEA^ detection has also been successfully used in a Thai clinical program [28]. However, RPA-based assays may require gel electrophoresis for result interpretation, whereas LAMP usually requires a higher reaction temperature and can be affected by carryover contamination from amplified DNA, which may lead to false-positive results. In this study, CRISPR-Cas12a was combined with RPA to provide sequence-specific target recognition and allow visual detection using the naked eye and lateral-flow assays without the need for specialized equipment.

Herein, we developed a novel diagnostic platform, gap-recombinase polymerase amplification combined with CRISPR-Cas12a (gap-RPA/CRISPR-Cas12a), for the rapid and accurate screening of both the --^SEA^ and --^THAI^ deletions. This practical, reliable, and accessible tool can be deployed in resource-limited settings and at the point-of-care, thereby expanding the reach of genetic screening and improving public health outcomes for populations at risk of α-thalassemia.

## 2. Materials and Methods

### 2.1. Genomic DNA Preparation and Genotype Confirmation

Genomic DNA samples used in this study were generously provided by the Thalassemia Research Unit, Faculty of Medicine, Naresuan University. The total sample set comprised 74 individuals: 26 with the --^SEA^ deletion, 18 with the --^THAI^ deletion, and 30 wild-type samples, extracted using the PureLink™ Genomic DNA Mini Kit (Invitrogen; Thermo Fisher Scientific, Inc., Waltham, MA, USA). Conventional real-time PCR was conducted according to Ruengdit et al. (2023) [29] and employed as the gold standard assay for genotype confirmation. The SGE1F/SGE2R/SGE3R primer system was used to identify the --^SEA^ deletion and normal allele, with the T1-F/T2-R primer pair applied for detection of the --^THAI^ deletion (Table 1).

### 2.2. Design of Single-Stranded DNA Amplification via RPA

RPA primers were designed based on the α-globin gene sequence retrieved from the NCBI database (accession no. NC_060940.1) and optimized according to RPA requirements with primer lengths of 30–35 nucleotides. Sequence alignment and primer region selection were performed using Multalin software 5.4.2 (http://multalin.toulouse.inra.fr (accessed on 15 December 2024); [30]). Primers for the detection of the normal allele were designed to amplify the *HBA2* region, and primers for gap detection of the --^SEA^ and --^THAI^ deletions were designed to anneal to sequences flanking the known breakpoint regions. For the --^THAI^ deletions, a naturally occurring PAM (TTTV) sequence was identified within the target region and used for Cas12a recognition. For the --^SEA^ target, no suitable natural PAM sequence was available within the selected target region. Therefore, the RPA primer was modified to introduce a synthetic PAM (TTTV) sequence into the RPA amplicon. The modified primer was designed to generate an amplicon containing the required PAM sequence for subsequent recognition by the Cas12a–crRNA complex. The gRNA-F and gRNA-R sequences were designed to generate gRNA targeting each amplicon. The design was performed using Multalin software to align and identify a suitable PAM site (TTTV) and a subsequent target sequence of 18–20 nucleotides.

### 2.3. Selection of Optimal RPA Primers

The designed primer sets were screened to identify the most efficient combination for the RPA reactions performed using the TwistAmp Basic Kit (TwistDx, Cambridge, UK) according to the manufacturer’s instructions. A 47.5 μL reaction mixture was prepared, consisting of 29.5 μL of dehydration-free buffer, 2.4 μL each of 10 μM Forward and Reverse primers, 12.2 μL of nuclease-free water, 1 μL of template DNA, and 2.5 μL of 280 mM Mg(CH_3_COO)_2_. The mixture was briefly vortexed and incubated at 37 °C for 60 min. The amplification products were analyzed on a 1.5% agarose gel stained with 2.5 µL ethidium bromide using 1× TAE buffer, with TrackIt^TM^ 1 kb Plus DNA Ladder (Invitrogen; Thermo Fisher Scientific, Inc., Waltham, MA, USA) as the size marker. Electrophoresis was carried out at 80 volts for 40 min to determine the most suitable RPA primer combination.

### 2.4. gRNA Synthesis for CRISPR-Cas12a

gRNA was synthesized in vitro using a HiScribe™ T7 Quick High Yield RNA Synthesis Kit (New England Biolabs, Hitchin, UK). The reaction mixture, with a total volume of 30 μL, contained 8 μL of nuclease-free water, 10 μL of 20 mM NTP Buffer Mix, 1 µg of gRNA-F and -R (Table 1), and 2 μL of T7 RNA Polymerase Mix. The mixture was incubated at 37 °C for 16 h. Following incubation, 2 μL of DNase I and 20 μL of distilled water were added, and the mixture was incubated at 37 °C for an additional 15 min. The synthesized gRNA was then purified using a Monarch RNA Cleanup Kit (New England Biolabs, Hitchin, UK). The purification protocol involved adding 100 μL of RNA cleanup binding buffer and 150 μL of 95% ethanol to 50 μL of the gRNA sample. This mixture was then loaded onto a column, centrifuged at 13,000 rpm for 1 min, washed with 500 μL of RNA cleanup wash buffer, and centrifuged again. The purified gRNA was then eluted with 20 μL of nuclease-free water.

### 2.5. Optimization of RPA Experimental Conditions

Each RPA reaction was optimized to determine the ideal temperature and incubation time, and performed using the TwistAmp Basic Kit (TwistDx, Cambridge, UK) as described previously. The reactions were incubated at 37 °C, 39 °C, and 42 °C for 10–60 min. The amplification products were analyzed using 1.5% gel electrophoresis in 1× TAE buffer at 80 volts for 40 min and visualized under a UV transilluminator to classify the optimal conditions.

### 2.6. CRISPR–Cas12a-Mediated RPA Product Detection

To detect the RPA amplicons, a CRISPR-Cas12a detection system consisting of Cas12a (New England Biolabs, Hitchin, UK and a synthesized gRNA was employed. A complex of Cas12a and gRNA was prepared by mixing 3 μL of 10X NEB buffer 1.2, 3 μL of 1 μM Cas12a, and 300 nM of guide RNA. This mixture was incubated at 37 °C for 10 min. Subsequently, 1 μL of 50 pmol ssDNA reporter (Table 1) and 5 μL of the RPA product were added. The reaction was incubated at 37 °C for 30 min, and the resulting fluorescence was observed under a UV transilluminator. The analytical limit of detection and minimum detectable DNA input were evaluated using a 10-fold serial dilution of genomic DNA. The dilution started at 10 ng and was continued to obtain eight concentration levels (10^1^–10^−6^). Each concentration was tested by RPA amplification followed by CRISPR-Cas12a detection under the optimized conditions. To evaluate cross-reactivity, the assay was tested using samples carrying clinically relevant α-globin variants. The samples included large deletional variants, including --^SEA^, --^THAI^, α^−3.7^, and α^−4.2^, as well as non-deletional point mutations such as Hb Constant Spring and Hb Pakse.

### 2.7. Assay Sensitivity and Specificity for α-Thalassemia Detection

To systematically validate the diagnostic performance of the developed gap-RPA/CRISPR-Cas12a assay, the optimized protocol was tested across all 74 clinical genomic DNA samples. The clinical evaluation comprised 222 target analyses, resulting from the independent testing of 74 samples against three distinct genetic markers. The assay’s diagnostic capabilities were determined by comparing the visual fluorescence readouts of our developed platform against the reference genotypic data obtained from the gold standard real-time PCR method. The diagnostic parameters, including sensitivity, specificity, positive predictive value (PPV), negative predictive value (NPV), and accuracy, were calculated using the standard epidemiological formulas below [31]. The *HBA2* reaction was used as an internal amplification control to confirm the presence of amplifiable DNA. Samples with negative *HBA2* and negative deletion-specific reactions were considered invalid and were repeated using the complete three-reaction panel. When *HBA2* amplification failed but a deletion-specific signal was detected, the sample was also retested to confirm the result. The final interpretation was based on the repeat test, and samples that remained discordant after repeat testing were considered invalid.

Sensitivity (%) = TP/(TP + FN) × 100

Specificity (%) = TN/(TN + FP) × 100

Positive Predictive Value (PPV, %) = TP/(TP + FP) × 100

Negative Predictive Value (NPV, %) = TN/(TN + FN) × 100

Accuracy (%) = (TP + TN)/(TP + TN + FP + FN) × 100

The true positives (TPs) and true negatives (TNs) represented the number of samples correctly identified by our assay in agreement with the real-time PCR. Conversely, false positives (FPs) and false negatives (FNs) denoted discordant results where the assay incorrectly yielded a positive or negative signal compared with the gold standard.

### 2.8. Screening to Detect the α^0^-Thalassemia Trait Using Gap-RPA/CRISPR-Cas12a as a Field Test

The primary objective of this study was to develop a diagnostic tool suitable for decentralized POC diagnosis. To evaluate the field applicability of the assay, the full gap-RPA/CRISPR-Cas12a workflow was conducted using a portable heat block for amplification, with detection using the naked eye. The accuracy and practical utility of the assay were evaluated using a blinded panel of 10 samples with known genotypes, including --^SEA^ deletion, --^THAI^ deletion, and wild-type (WT) controls. The potential for use in minimally instrumented POC settings was evaluated by extracting DNA from non-invasive specimen types, including hair follicles and buccal swabs. The performance of two simple DNA extraction methods, namely the HotSHOT method [32] and the Universal All Tissue Extraction Kit (Yeastern Biotech Co., Ltd., Taipei, Taiwan), was compared. The efficiency of these methods was evaluated using 1, 2, and 3 hair follicles per sample as source material, and 10 and 20 buccal swabs per sample. The assay was designed to be performed using minimal equipment, such as a portable heating block for the RPA amplification and a simple UV pad for the CRISPR-Cas12a readout.

The results of the gap-RPA/CRISPR-Cas12a workflow were also analyzed using a lateral flow dipstick assay (Milenia HybriDetect; Milenia Biotec., Frankfurt, Germany). A 100 μL volume of the HybriDetect assay buffer was mixed with 10 μL of the RPA/CRISPR-Cas12a product. The mixture was briefly vortexed, and a lateral flow dipstick was dipped into the solution at the sample pad end. After a 10 min incubation to allow for capillary action to move the solution to the absorbent pad, the results were read from the test strip. Our developed diagnostic system was then evaluated using two distinct readout platforms: gap-RPA/CRISPR-Cas12a via fluorescence and gap-RPA/CRISPR-Cas12a via a lateral flow assay (LFA). The accuracy of our readout platforms was evaluated using the same blinded test samples. To approximate field conditions, the procedures were performed by users with no formal research training. The accuracy of each detection method was calculated by comparing its results to the confirmed genotypes obtained from the real-time PCR standard.

## 3. Results

### 3.1. Conventional PCR for Genotyping

**Figure 1:**
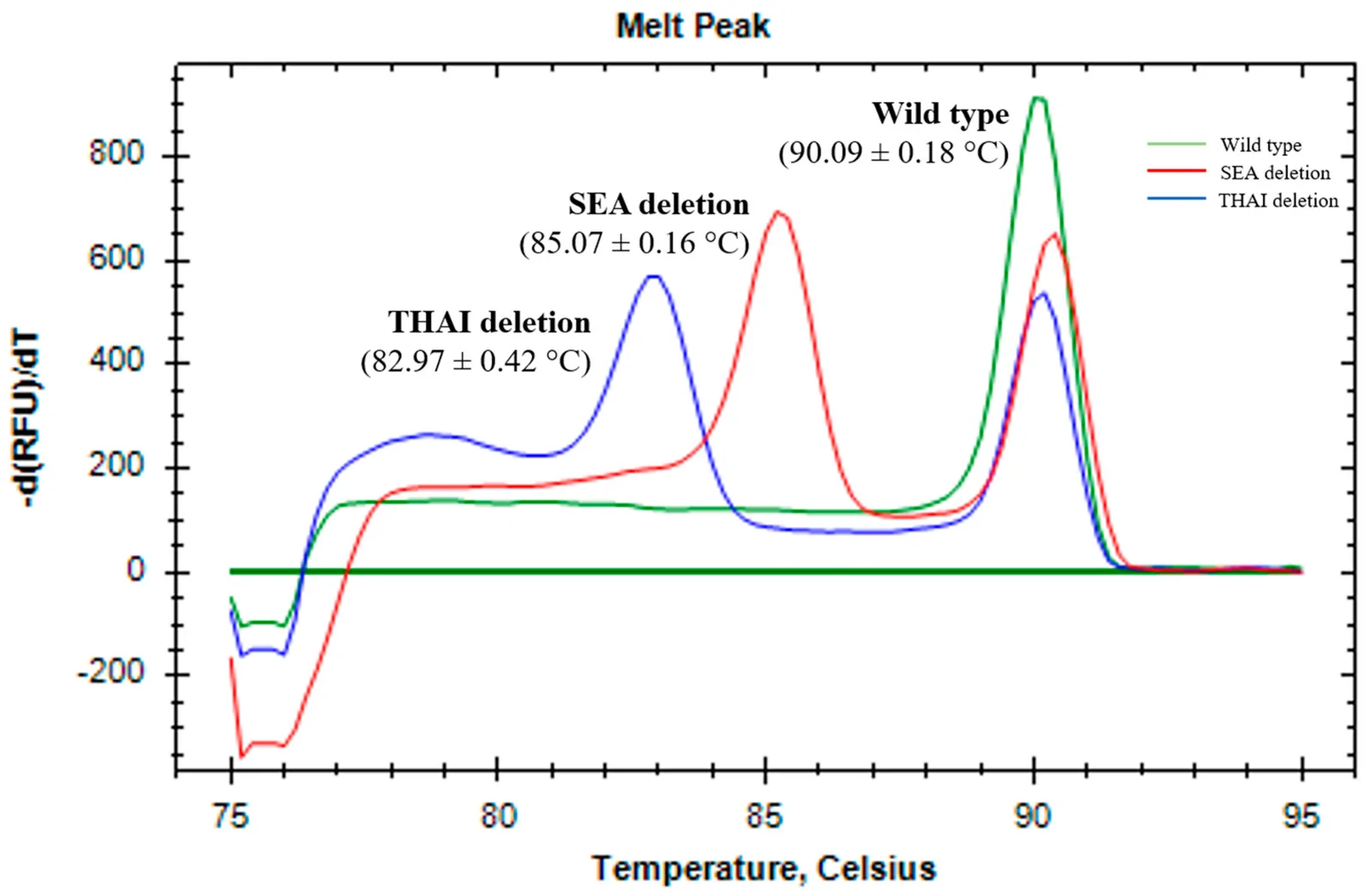
Representative melting peak profiles of wild type, --^SEA^, and --^THAI^ heterozygotes.

Real-time PCR amplification was conducted on samples from individuals with the α^0^-thalassemia --^SEA^, --^THAI^ deletion and from normal, wild-type individuals. The amplified fragments from the wild type, --^SEA^ and --^THAI^ deletions had specific peaks at melting temperatures (Tm) of 90.09 ± 0.18 °C, 85.07 ± 0.16 °C and 82.97 ± 0.42 °C, respectively (Figure 1: Supplementary Table S1).

### 3.2. Design and Selection of Primers for RPA Amplification

**Figure 2:**
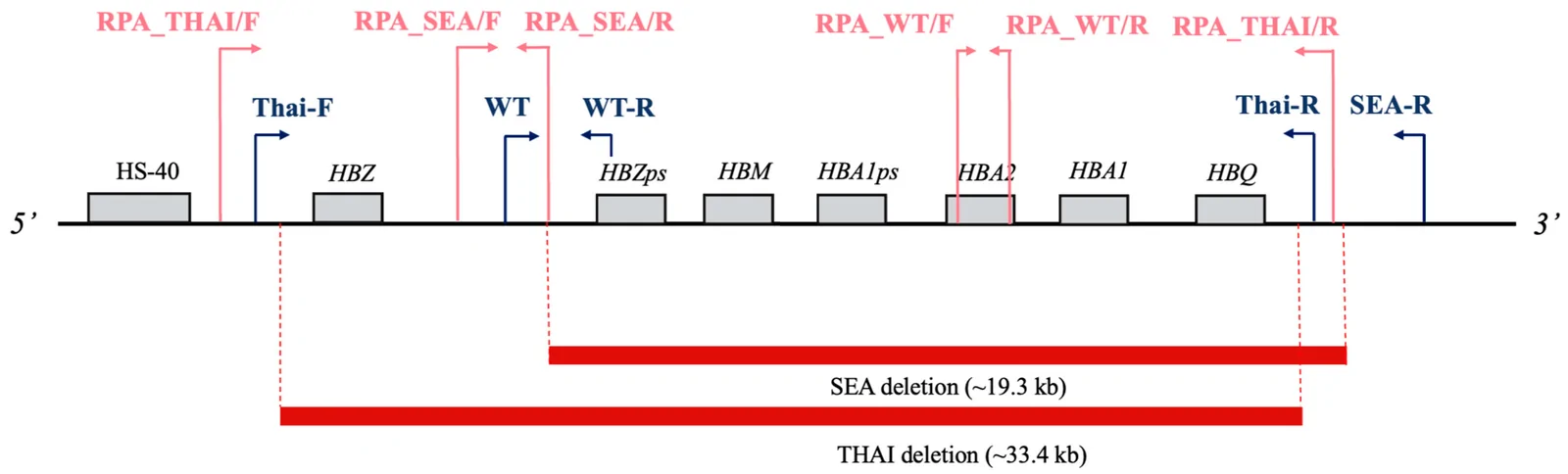
Primer set selection suitable for the RPA reaction. Schematic representation of the --^SEA^ (19.3 kb) and --^THAI^ (33.4 kb) deletions. The diagram is not drawn to scale.

The RPA primers were specifically designed to flank the deletion breakpoints of the α-globin gene (Figure 2). Importantly, these target regions must include a TTTV protospacer adjacent motif site, which is strictly required for CRISPR-Cas12a recognition. To determine the most effective primer pairs, various sets were evaluated by incubating the RPA reactions at 37 °C for 1 h. Subsequent analysis via 1.5% agarose gel electrophoresis demonstrated that the *HBA2* and --^THAI^ primer sets produced specific products at their expected sizes of 222 bp and 234 bp, respectively (Supplementary Figure S1). Because the native breakpoint region of the --^SEA^ deletion lacks a naturally occurring PAM site, three modified primer combinations were specifically designed and assessed to overcome this limitation. Among these, the SEA-F and SEA-R2 primer set yielded a strong band at the expected size of 188 bp, with no non-specific amplification observed (Supplementary Figure S2). DNA sequencing analysis confirmed that the selected modified primer sets contained the expected nucleotide sequences and the intended modification sites (Supplementary Figure S3).

### 3.3. Optimization of RPA Reaction Conditions

The optimal conditions for the RPA reaction were determined at 37 °C, 39 °C, and 42 °C. Previous studies indicated that the optimal temperature for RPA was typically within the 37–42 °C range, but our results indicate clear differences in amplification efficiency and specificity across this range. At 37 °C, a clear DNA band at the expected size of 188 bp was observed within the first 10 min. At 39 °C, a DNA band at 188 bp was also observed within 10 min, but the band was noticeably fainter compared with the 37 °C reaction. At 42 °C, no target band was visible at 10 min; a faint 188 bp band began to appear at 20 min, and as the reaction time increased, more prominent non-specific bands (unspecific bands) were observed. These findings confirmed 37 °C as the optimal temperature for our specific assay, balancing both speed and specificity.

### 3.4. CRISPR-Cas12a-Based Detection

**Figure 3:**
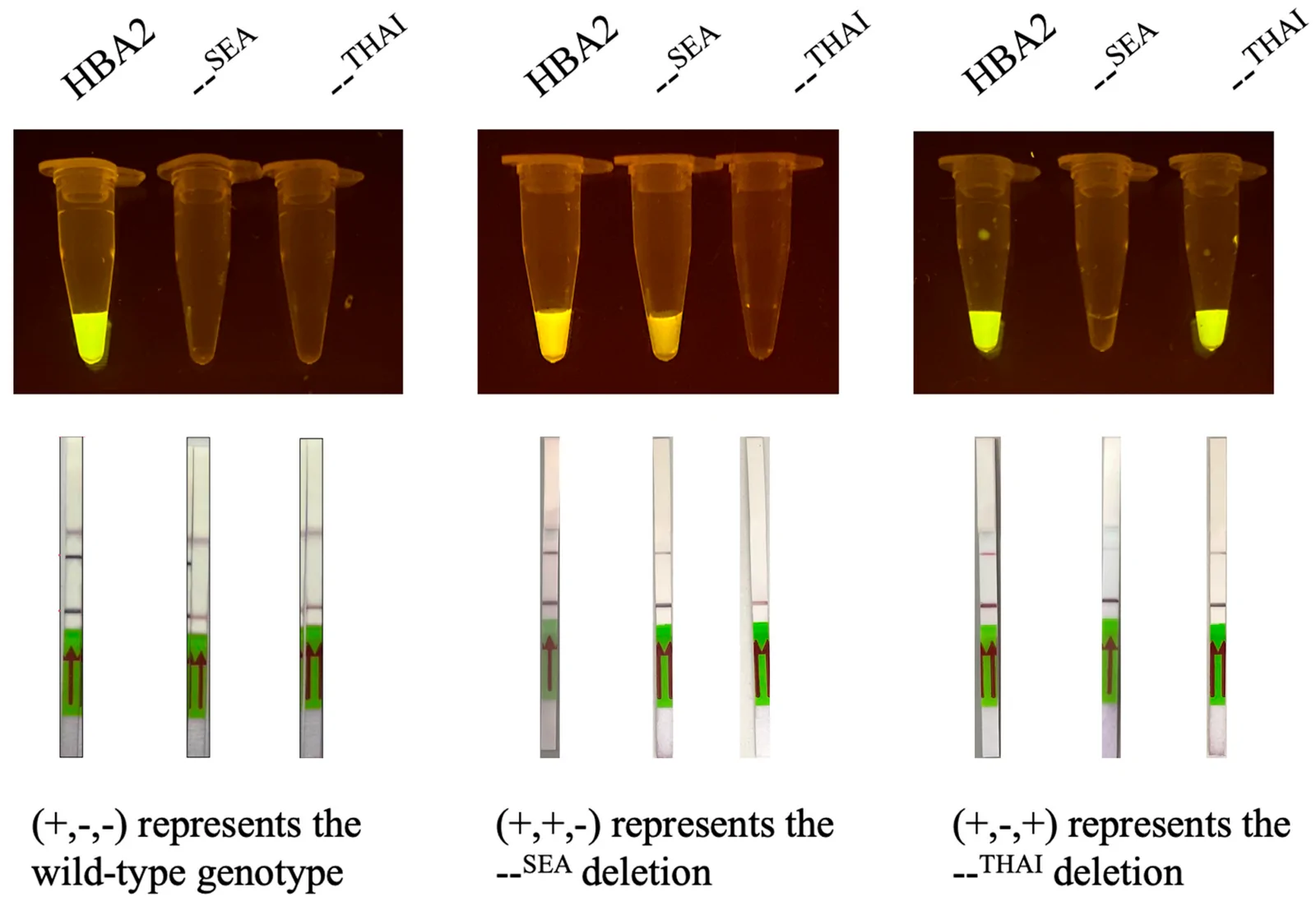
Representative validation results of the RPA-CRISPR/Cas12a assay tested with different primer sets in the wild type, heterozygous for --^SEA^ deletion, and heterozygous for --^THAI^ deletion.

The final step of our developed diagnostic system involved the detection of RPA amplicons using the CRISPR-Cas12a platform, with results visually analyzed under blue light illumination. Quantitative fluorescence measurements showed clear differences in signal intensity between positive and negative reactions of the gap-RPA/CRISPR-Cas12a assay (Supplementary Figure S4). In this assay, the collateral cleavage activity of Cas12a upon target recognition degrades a fluorescent reporter probe, generating a strong signal visible to the naked eye. As illustrated in Figure 3, each sample was evaluated using a three-tube panel: *HBA2* (serving as an indicator for the normal allele), the --^SEA^ deletion, and the --^THAI^ deletion. For the wild-type genotype, only the *HBA2* reaction exhibited bright fluorescence, yielding a distinct (+, −, −) visual readout. By contrast, heterozygous samples display dual fluorescence, indicating the presence of both a normal allele and a specific deletion. Specifically, a heterozygote with the --^SEA^ deletion generates fluorescence in both the *HBA2* and --^SEA^ tubes (+, +, −). Similarly, a heterozygote with the --^THAI^ deletion produces positive fluorescent signals in both the *HBA2* and --^THAI^ reaction tubes (+, −, +). To evaluate analytical specificity, the assay was tested against clinically relevant α-globin variants. The panel comprised large deletional variants (--^SEA^, --^THAI^, α^−3.7^, and α^−4.2^) and non-deletional point mutations including Hb Constant Spring and Hb Pakse. No cross-reactivity was observed with the non-target variants (Supplementary Figure S5). Furthermore, the analytical sensitivity was evaluated using serially diluted DNA templates. RPA products were detectable by agarose gel electrophoresis down to 10^−2^ ng, whereas the RPA/CRISPR-Cas12a assay detected DNA down to 10^−3^ ng. Notably, a positive CRISPR-Cas12a signal was still observed at DNA concentrations where the corresponding RPA product was no longer visible on the agarose gel (Supplementary Figure S6: Supplementary Table S2). This indicates that the CRISPR-Cas12a detection step improved the sensitivity of low-level amplicon detection beyond conventional gel-based visualization.

The assay time was calculated from the point at which extracted DNA was available for amplification. DNA preparation time is determined by the specimen type and the extraction method employed. RPA amplification and Cas12a–gRNA pre-incubation were performed in parallel at 37 °C for 10 min. The RPA product was then added to the CRISPR-Cas12a reaction and incubated for 30 min for target recognition and reporter cleavage. Fluorescence results were read directly after the CRISPR reaction, giving a total assay time of approximately 40 min from extracted DNA to fluorescence readout. LFA detection required an additional 10 min, giving a total assay time of approximately 50 min from extracted DNA to the final result (Supplementary Figure S7).

To determine the clinical applicability of the developed platform, diagnostic accuracy was evaluated in comparison with established reference methods using a panel of 74 blinded samples. Gap-RPA/CRISPR-Cas12a assays were carried out by personnel without prior research training. As presented in Table 2, conventional amplification approaches, including real-time PCR and RPA, demonstrated optimal performance, achieving 100% accuracy across all tested genotypes. These included the internal control *HBA2*, the --^SEA^ deletion, and the --^THAI^ deletion. The gap RPA, coupled with the CRISPR-Cas12a assay using fluorescence detection, also demonstrated strong diagnostic performance, although sensitivity varied among targets. The clinical performance of the gap-RPA/CRISPR-Cas12a assay was evaluated using 74 genomic DNA samples, with results compared against the gold standard real-time PCR method (Table 2). The developed assay demonstrated complete concordance with real-time PCR for the --^THAI^ deletion, correctly identifying all 18 positive and 56 negative samples (100% sensitivity and specificity). For the --^SEA^ deletion, the assay correctly detected 25 of 26 positive cases and all 48 negative cases, resulting in a sensitivity of 96.15% and a specificity of 100%**.** Detection of the wild-type target showed a sensitivity of 90% (27/30 positive samples) and a specificity of 100% (44/44 negative samples). The assay yielded no false-positive results across any of the targets, maintaining a positive predictive value (PPV) of 100%. When combining the data from all 222 target tests, the sensitivity and specificity were 94.59% and 100%, respectively, yielding a total diagnostic accuracy of 98.20% (Table 3).

The reliability of simplified DNA extraction protocols requiring minimal equipment was also assessed, as summarized in Table 4 (*N* = 10). The HotSHOT protocol was compared with a commercial Universal All Tissue Extraction Kit using noninvasive biological materials, specifically hair follicles and buccal swabs. Extraction efficiency was assessed using two downstream detection formats: gap-RPA/CRISPR-Cas12a via fluorescence readout and gap-RPA/CRISPR-Cas12a via lateral flow. The detection platforms produced fully consistent results, demonstrating that extraction performance was not affected by the readout format (80%). The HotSHOT protocol demonstrated high reliability, achieving 100% accuracy across all sample types and input quantities. Successful DNA recovery was obtained from minimal material, with one to three hair follicles and ten to twenty buccal swabs.

By contrast, the Universal All Tissue Extraction Kit performed well with hair follicle samples, maintaining 100% accuracy regardless of follicle number, but performance decreased when applied to buccal swabs. For ten-swab and twenty-swab samples, accuracy declined to 80% for both fluorescence and lateral flow assay detection. These results support the HotSHOT protocol as a versatile method for rapid DNA extraction from non-invasive samples, providing DNA templates of sufficient quality for CRISPR-Cas12a-based detection.

## 4. Discussion

Severe α^0^-thalassemia deletions, such as --^SEA^ and --^THAI^, can lead to fatal conditions like Hb Bart’s hydrops fetalis, making rapid and accessible carrier screening essential [8]. This study developed a novel diagnostic platform as gap-recombinase polymerase amplification combined with CRISPR-Cas12a (gap-RPA/CRISPR-Cas12a) for the rapid and accurate screening of the α^0^-thalassemia Southeast Asian (--^SEA^) and Thai (--^THAI^) deletions. Designing a suitable PAM for CRISPR-Cas12a detection of α^0^-thalassemia deletions can be challenging, particularly for GC-rich regions of the α-globin gene. The absence of an appropriate natural PAM in the --^SEA^ target therefore required an alternative primer engineering strategy to introduce a synthetic PAM for Cas12a recognition. The SEA-F and SEA-R2 primer set was selected because no non-specific amplification was observed, indicating high amplification specificity. Sequence analysis further confirmed the expected target sequence, supporting the reliability of this primer set for specific detection of the --^SEA^ deletion. Our clinical evaluation demonstrated exceptional reliability, achieving 100% specificity and 98.20% accuracy across 222 target evaluations. However, four false-negative results were observed, prompting an investigation into the assay’s limitations. The *HBA2* reaction served as an internal amplification control to confirm the presence of amplifiable DNA. Failure of the *HBA2* reaction may indicate insufficient or degraded DNA and can complicate the interpretation of negative deletion results. Therefore, samples with failed *HBA2* amplification should be retested before final interpretation. These false-negative results were primarily attributed to pre-analytical factors, specifically sample quality and operator-induced effects during the manual DNA extraction process [33,34,35]. When using a commercial DNA extraction kit, the final DNA yield heavily depended on the initial quality of the clinical specimen. Factors such as insufficient blood collection volume, the presence of micro-clots, or DNA degradation due to improper sample storage can lead to a critically low concentration of starting genetic material [36,37]. If the input DNA is below the amplification threshold, the gap-RPA step fails to generate enough target amplicons [33,37]. Consequently, without a sufficient concentration of amplified targets, the Cas12a-crRNA complex cannot be fully activated to cleave the reporter, resulting in an absent fluorescent signal. The results demonstrate that this integrated system was a viable and powerful tool, offering significant advantages over conventional methods for POC applications. Our initial findings confirmed the genotypes of the samples using conventional gap-PCR and aligned with the established literature [2]. This foundational step was crucial as it provided a reliable gold standard to validate the performance of our novel assay. The successful design and optimization of our RPA primers and gRNA were central to the success of this platform. Our specific primer set amplified the target region within 10 min at the low temperature of 37 °C. Our assay can be performed with minimal equipment and a simple heat block, eliminating the need for a sophisticated thermal cycler. The optimal temperature of 37 °C reduced the equipment requirements and also minimized the risk of non-specific amplification, as evidenced by the increase in unspecific bands at higher temperatures (42 °C). This rapid, low-temperature amplification is a key advantage of our gap-RPA assay over traditional PCR and other isothermal methods that require higher, less stable temperatures. The integration of CRISPR-Cas12a as a detection method provided the assay with exceptional specificity and a user-friendly readout [20,21,22]. As demonstrated by our results, the gRNA, designed to target the RPA amplicon, enabled the Cas12a enzyme to specifically recognize and cleave its target, thereby triggering its potent trans-cleavage activity. The resulting fluorescent signal was clearly visible in positive samples within a short time frame, validating the high sensitivity of the system. This principle, which has been successfully applied in the detection of various pathogens like the Zika virus [38] and SARS-CoV-2 [23] are well-suited for genetic screening [39,40].

One of the most compelling aspects of our developed gap-RPA/CRISPR-Cas12a assay is its significant economic advantage and cost-effectiveness compared with conventional PCR-based methods [20,21,22,39,40]. The initial setup for a PCR laboratory requires substantial capital investment for thermal cyclers, centrifuges, and gel electrophoresis systems, but our assay can be performed with a simple heating block or even a portable device [41]. This drastically reduces infrastructure costs, making the technology highly accessible to smaller hospitals, local health clinics, and rural communities that lack the financial resources for sophisticated molecular diagnostics. The reagents for RPA and CRISPR-Cas12a are relatively inexpensive and can be stored at ambient temperatures, reducing the need for cold-chain logistics in the field [42,43]. The lateral flow dipstick serves as a low-cost, disposable readout that provides a clear positive or negative result without the need for expensive imaging systems or complex data analysis [44,45]. Similarly, a parallel study successfully developed a colorimetric LAMP assay to detect point mutations, including Hb CS and Hb PS, achieving 100% sensitivity and specificity [46]. A comparison of these two isothermal platforms revealed complementary diagnostic strengths. Our gap-RPA/CRISPR-Cas12a assay operates at 37 °C and is highly efficient for capturing large deletions like --^SEA^ and --^THAI^. Conversely, the LAMP assay operates at 65 °C and utilizes a visual pH-based color change [46,47] (Supplementary Table S3). The platform meets several aspects of the ASSURED framework, including rapid detection, high specificity, simple operation, and visual LFA readout. However, basic equipment and manual steps are still required. Further work is needed to simplify the workflow, assess operator reproducibility and field performance, and develop reagent lyophilization [48]. The *HBA2* reaction served as an internal amplification control, and failed reactions should be repeated before final interpretation. The ability to obtain a result within 40 min, from sample to readout, also represents a major practical benefit. This rapid turnaround time enables immediate genetic counseling for at-risk couples, facilitates timely prenatal diagnosis decisions, and prevents the need for patients to make multiple trips to a centralized laboratory. The simplicity of the protocol, combined with the ease of result interpretation via the lateral flow dipstick assay, ensures that healthcare workers with minimal molecular biology training can effectively perform the test, further enhancing its usability and reducing the reliance on highly specialized personnel.

A major strength of our developed system is its adaptability for POC applications, as confirmed by the successful lateral flow dipstick analysis. This visual readout method provides a clear, unmistakable result that can be interpreted by a layperson without specialized equipment, making it ideal for use in small clinics, community health centers, or even home settings. The positive two-band result for the target deletion contrasts with the negative single-band result for non-target samples. The average cost of traditional PCR is 1.58 USD per sample [49]. The RPA/CRISPR-Cas12a assay costs approximately USD 2.58 per sample with fluorescence readout. An additional USD 1.50 is required for LFA, resulting in a total cost of approximately USD 4.08 per test [50]. Thus, the reagent cost of the present assay is higher than that of conventional PCR. However, the assay requires less complex instrumentation and does not require a thermal cycler or gel electrophoresis for result interpretation, which may reduce equipment- and workflow-related costs. The reported LAMP strategy reduced diagnostic costs by approximately USD 2608 for 210 samples through shorter processing time, lower workload, and reduced dependence on advanced equipment [28]. Similarly, our gap-RPA/CRISPR-Cas12a platform may offer practical savings in equipment use and workflow time. Our protocol has potential for non-invasive sampling using the simple DNA extraction method, thereby eliminating the need for expensive commercial extraction kits. This integrated platform reduces the reliance on expensive thermal cyclers and gel electrophoresis systems, making our gap-RPA/CRISPR-Cas12a platform highly cost-effective and an ideal point-of-care testing tool for rapid α0-thalassemia screening in resource-limited settings. Future studies should focus on creating a multiplex assay capable of simultaneously screening for both deletions in a single reaction. A non-instrumental DNA extraction method is also essential to create a complete and field-ready POC system.

## 5. Conclusions

This study highlighted the potential of the gap-RPA/CRISPR-Cas12a platform as a practical diagnostic tool for α^0^-thalassemia screening, particularly in resource-limited regions where conventional molecular methods are impractical. The full workflow provides a simplified and highly accurate screening approach for both --^SEA^ and --^THAI^ deletions. By integrating an easy-to-interpret visual readout with the ability to safely process non-hazardous samples, this diagnostic method is ideal for practical field-level applications. Our results confirm that the platform can deliver reliable, rapid, and highly specific detection of clinically critical thalassemia deletions under isothermal conditions using minimal equipment. Future applications of this approach can support broader accessibility of molecular diagnostics in low- and middle-income countries.

## Figures and Tables

**Table 1:** Single-stranded nucleotides utilized in this research.

Primer	Sequence (5′ → 3′)	Protocol
^1^ WT	AGAAGCTGAGTGATGGGTCCG	To detect --^SEA^ and --^THAI^ as conventional method
^1^ WT-R	ACAAACGCCCGTCCGACTCAA	
^1^ SEA-R	TGGACTTAAGTGATCCTCCTGCCC	
^1^ Thai-F	ATTCACATACCATACGGTTCAC	
^1^ Thai-R	AATTCCCCTGGACTTGAGTG	
^2^ RPA_WT/F	TGACCCTCTTCTCTGCACAGCTCCTAAGCCAC	The internal control sets
^2^ RPA_WT/R	GCCGGTGCAAGGAGGGGAGGAGGGCCCGTTGG	
^3^ gRNA_WT/F	CTAATACGACTCACTATAGGGTTTCAAAGATTAAATAATTCCCACTAAGTGTGGGTGAGGTCAGCACGGTGCTCACAGAA	
^3^ gRNA_WT/R	TTCTGTGAGCACCGTGCTGACCTCACCCACACTTAGTGGGAATTATTTAATCTTTGAAACCCTATAGTGAGTCGTATTAG	
^2^ RPA_SEA/F	GCGATCTGGGCTCTGTGTTCTCAGTATTGGAG	To detect --^SEA^ by RPA combined CRISPR-Cas12a
^2^ RPA_SEA/R1F	GTGGTCCTCCTGCCCCAGCCTCCATTTGAACC	
^2^ RPA_SEA/R2F	GTGGTCCTCCTGCCCCAGCCTTTAAGTGAACC	
^2^ RPA_SEA/R3F	GTGGTCCTCCTGCCCCAGTTTCCAAGTGAACC	
^3^ gRNA_SEA/F	CTAATACGACTCACTATAGGGTTTCAAAGATTAAATAATTCCCACTAAGTGTGGGTAAGTGAACCTCCCCAAGGCGC	
^3^ gRNA_SEA/R	GCGCCTTGGGGAGGTTCACTTACCCACACTTAGTGGGAATTATTTAATCTTTGAAACCCTATAGTGAGTCGTATTAG	
^2^ RPA_Thai/F	GGAAGAATAAAGCGAGAGGAATCACATTCCTC	To detect --^THAI^ by RPA combined CRISPR-Cas12a
^2^ RPA_Thai/R	CCCTGGACTTGAGTGGGCATGAGTCTCCCCAA	
^3^ gRNA_Thai/F	CTAATACGACTCACTATAGGGTTTCAAAGATTAAATAATTCCCACTAAGTGTGGGTAAGTGTATAATTCAGGCCGGGCG	
^3^ gRNA_Thai/R	CGCCCGGCCTGAATTATACACTTACCCACACTTAGTGGGAATTATTTAATCTTTGAAACCCTATAGTGAGTCGTATTAG	
^4^ ssDNA_reporter	56-FAM/TTATT/3IABkFQ	ssDNA for *trans* activated for fluorescence assay
^4^ ssDNA_reporter	56-FAM/TTATT/3Bio	ssDNA for *trans* activated for LFA assay

^1^ Nucleotide sequence used as a primer for PCR. ^2^ Nucleotide sequence used as a primer for RPA. ^3^ Nucleotide sequence used as a template for gRNA synthesis. ^4^ Nucleotide sequence used as a reporter for CRISPR-Cas12a detection.

**Table 2:** Diagnostic validation of the gap-RPA/CRISPR-Cas12a assay compared with the real-time PCR reference standard.

Target	Gap-RPA/CRISPR-Cas12a	Real-Time PCR		Total
		Positive	Negative	
Wild type	**Positive**	27	0	27
	**Negative**	3	44	47
	**Total**	30	44	74
SEA deletion	**Positive**	25	0	25
	**Negative**	1	48	49
	**Total**	26	48	74
Thai deletion	**Positive**	18	0	18
	**Negative**	0	56	56
	**Total**	18	56	74

**Table 3:** Evaluation of diagnostic metrics for the gap-RPA/CRISPR-Cas12a assay.

Target	Sensitivity (%)	Specificity (%)	PPV (%)	NPV (%)	Accuracy (%)
Wild type	90.00	100	100	93.62	95.95
Sea deletion	96.15	100	100	97.96	98.65
Thai deletion	100	100	100	100	100
Overall	94.59	100	100	97.37	98.20

**Table 4:** RPA/CRISPR-Cas12a assays from non-invasive samples using simple DNA extractions (*N* = 10).

	DNA Extraction Methods/Biological Samples	Hair Follicle Per Sample			Buccal Swab	
		1	2	3	10	20
gap-RPA/CRISPR-Cas12a via fluorescence	HotSHOT	100	100	100	100	100
	Univers All Tissue Extraction kit.	100	100	100	80	80
gap-RPA/CRISPR-Cas12a via LFA	HotSHOT	100	100	100	100	100
	Univers All Tissue Extraction kit.	100	100	100	80	80

## Data Availability

The original contributions presented in the study are included in the article; further inquiries can be directed to the corresponding author.

## Supplementary Materials

The following supporting information can be downloaded at: https://www.mdpi.com/article/10.3390/biology15181579/s1, Figure S1: Optimization of RPA reaction temperature (37–42 °C) using wild-type (WT), --^SEA^, and --^THAI^ primer sets; Figure S2: Agarose gel electrophoresis analysis of modified primer sets for the detection of the --^SEA^ deletion; Figure S3: DNA sequences of modified primer sets for the detection of the --^SEA^ deletion; Figure S4: Fluorescence intensity of the gap-RPA/CRISPR-Cas12a assay in representative genotypes: (A) wild type, (B) --^SEA^, and (C) --^THAI^. Data are presented as mean ± SD; Figure S5: Analytical specificity and cross-reactivity of the gap-RPA/CRISPR-Cas12a assay against clinically relevant α-globin variants; Figure S6: Analytical limit of detection of the gap-RPA/CRISPR-Cas12a assay using serially diluted genomic DNA; Figure S7: Schematic workflow and assay timing of the gap-RPA/CRISPR-Cas12a; Table S1: Melting temperatures (Tm) of 74 previously characterized DNA samples, including 30 wild-type, 26 Southeast Asian (--^SEA^) deletion, and 18 Thai (--^THAI^) deletion samples, determined by real-time PCR melting curve analysis; Table S2: Analytical sensitivity and limit of detection of the RPA/CRISPR-Cas12a assay at different DNA concentrations; Table S3: Comparison of analytical performance and practical requirements of isothermal assays for α^0^-thalassemia detection.
