# Supplementary material for: Development of Gap-Recombinase Polymerase Amplification Combined with CRISPR-Cas12a (Gap-RPA/CRISPR-Cas12a) for Rapid Screening of α0-Thalassemia Southeast Asian (--SEA) Deletion and Thai (--THAI) Deletion
Source: Biology (Basel). 2026 Sep 8;15(18):1579. doi: 10.3390/biology15181579 (PMC13603635; doi:10.3390/biology15181579)
Supplement: Supplementary file 1 [file biology-15-01579-s001.zip › biology-4464812-supplementary.pdf]

Supplementary Materials

Development of Gap-Recombinase Polymerase Amplification Combined with CRISPR-Cas12a (Gap-RPA/CRISPR-Cas12a) for Rapid Screening of α^0-Thalassemia Southeast Asian (--^{SEA}) Deletion and Thai (--^{THAI}) Deletion

Phanupong Changtor, Siriphat Muangpa and Nonglak Yimtragool *

Department of Biology, Faculty of Science, Naresuan University, Phitsanulok 65000, Thailand; phanupongc@nu.ac.th (P.C.); siriphatm64@nu.ac.th (S.M.)
* Correspondence: nonglakp@nu.ac.th

Table S1. Melting temperatures (Tm) of 74 previously characterized DNA samples, including 30 wild-type, 26 Southeast Asian (--^{SEA}) deletion, and 18 Thai (--^{THAI}) deletion samples, determined by real-time PCR melting curve analysis.

No.	Samples	Melting temperature (Tm, °C)		
		**THAI**	**SEA**	**WT**
41	SEA11	-	85.2	90.4
42	SEA12	-	85.2	90.2
43	SEA13	-	85.0	90.2
44	SEA14	-	85.0	90.2
45	SEA15	-	85.0	90.0
46	SEA16	-	85.0	90.0
47	SEA17	-	84.8	90.0
48	SEA18	-	84.8	90.0
49	SEA19	-	84.8	90.0
50	SEA20	-	84.8	90.0
51	SEA21	-	85.0	90.0
52	SEA22	-	85.0	90.2
53	SEA23	-	85.0	90.0
54	SEA24	-	85.0	90.0
55	SEA25	-	85.0	90.2
56	SEA26	-	85.0	90.0
57	THAI1	83.0	-	90.4
58	THAI2	83.0	-	90.4
59	THAI3	83.0	-	90.2
60	THAI4	82.8	-	90.2
61	THAI5	84.2	-	90.2
62	THAI6	83.8	-	90.2
63	THAI7	82.8	-	90.2
64	THAI8	83.0	-	90.2
65	THAI9	83.0	-	90.2
66	THAI10	82.8	-	90.2
67	THAI11	83.0	-	90.4
68	THAI12	82.8	-	90.2
69	THAI13	82.6	-	90.0
70	THAI14	82.6	-	90.2
71	THAI15	82.4	-	90.0
72	THAI16	82.6	-	90.4
73	THAI17	82.8	-	90.2
74	THAI18	83.2	-	90.4
	**X**	**82.97**	**85.07**	**90.09**
	**SD**	**0.42**	**0.16**	**0.18**

Table S2. Analytical sensitivity and limit of detection of the RPA/CRISPR-Cas12a assay at different DNA concentrations.

Concentration (ng)	RPA	CRISPR-Cas12a	Fluorescence intensity (Mean ± SD)	% Accuracy[1]
10^{1}	7/7	7/7	2901.13 ± 520.04	100
10^{0}	7/7	7/7	2492.76 ± 864.72	100
10^{-1}	7/7	7/7	2539.93 ± 344.50	100
10^{-2}	7/7	7/7	2517.73 ± 188.02	100
10^{-3}	0/7	7/7	2138.20 ± 575.50	100
10^{-4}	0/7	1/7	420.08 ± 743.00	14.28
10^{-5}	0/7	0/7	222.94 ± 17.91	0
10^{-6}	0/7	0/7	214.97 ± 6.27	0

[1]accuracy (%) was calculated based on the number of positive detections obtained using the gap-RPA/CRISPR-Cas12a assay relative to the total number of replicates tested.

Table S3. Comparison of analytical performance and practical requirements of isothermal assays for α^{0}-thalassemia detection.

Technique	Reaction temperature (°C)	Time	Sensitivity	Specificity	Sample type	Equipment requirements
LAMP-turbidimetric[28]	65	40	100	97.7	leftover-specimen	1.Thermal source eg. dry block heaters
RPA assay[27]	42 and 65	50 (excluding gel electrophoresis)	100	100	leftover-specimen	1.Thermal source eg. dry block heaters 2.gel electrophoresis 3. UV light
This study	37	40	94.59	100	leftover-specimen	1.Thermal source eg. dry block heaters

[] reference in main manuscript.

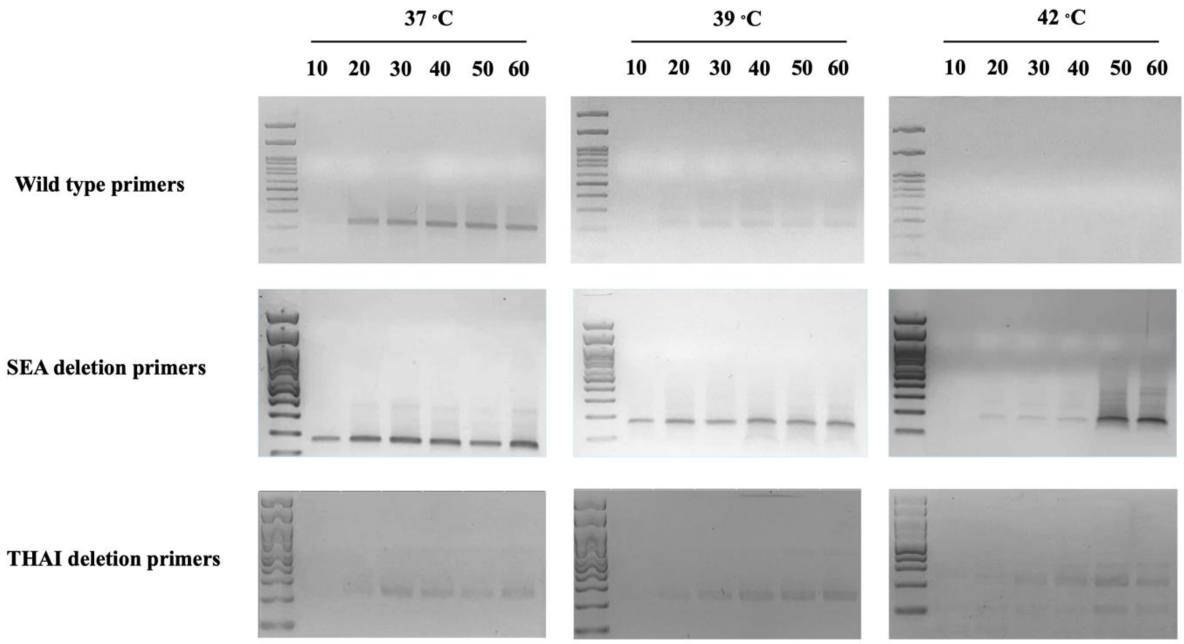

Figure S1. Optimization of RPA reaction temperature (37–42°C) using wild-type (WT), --^{SEA}, and --^{THAI} primer sets.

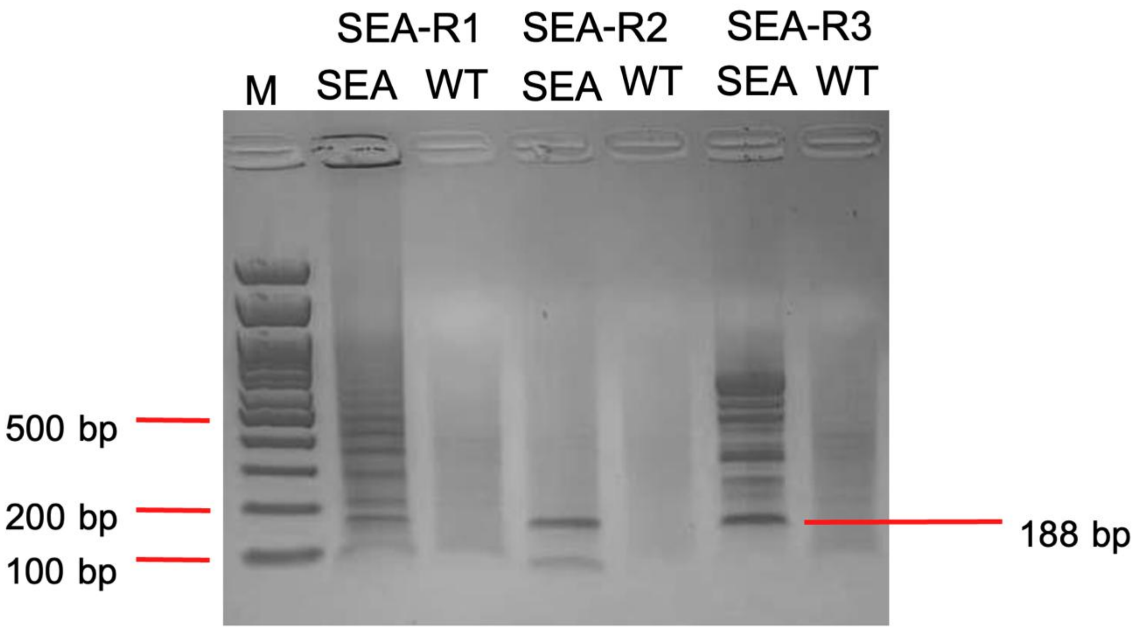

Figure S2. Agarose gel electrophoresis analysis of modified primer sets for the detection of the --^{SEA} deletion.

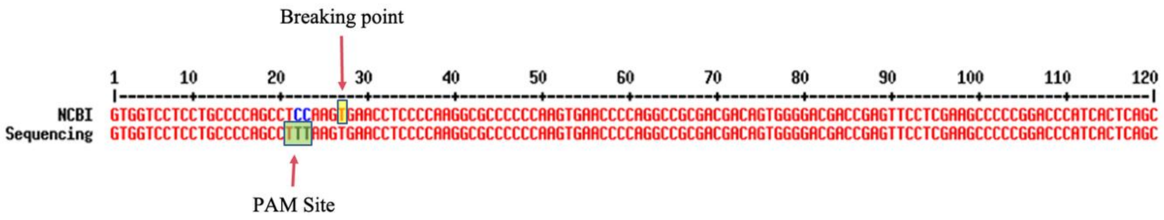

Figure S3. DNA sequences of modified primer sets for the detection of the --^{SEA} deletion.

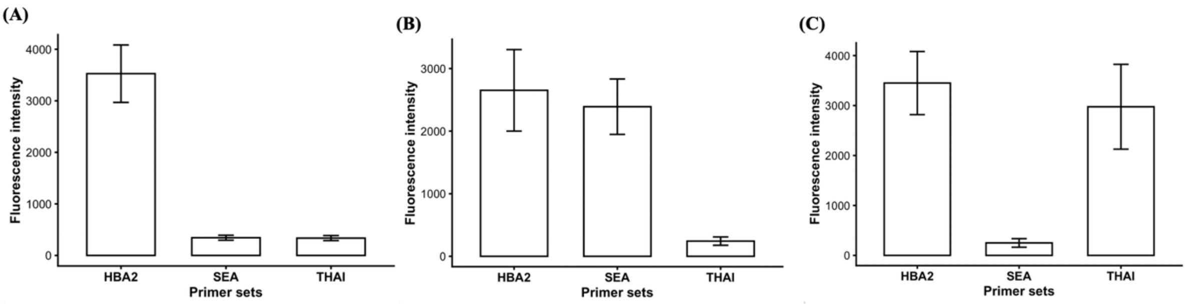

Figure S4. Fluorescence intensity of the gap-RPA/CRISPR-Cas12a assay in representative genotypes: (A) wild type, (B) --^{SEA}, and (C) --^{THAI}. Data are presented as mean ± SD.

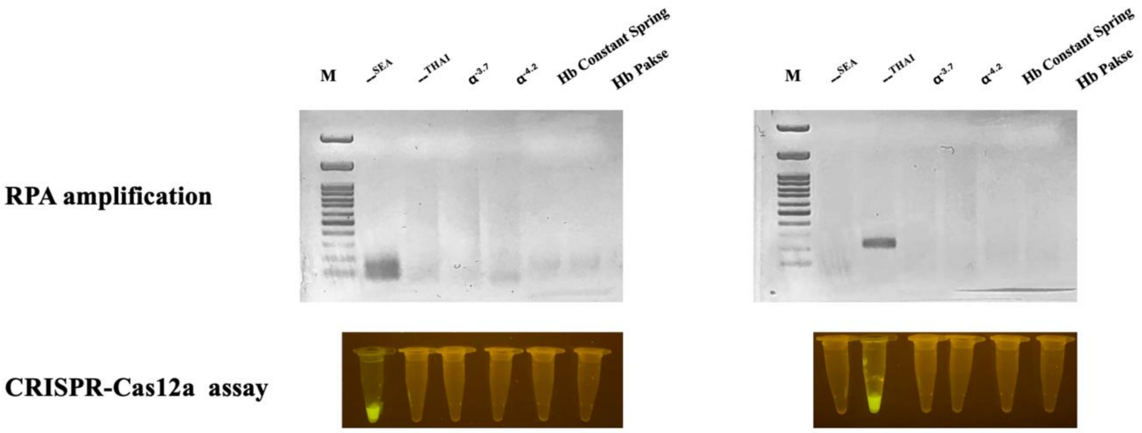

Figure S5. Analytical specificity and cross-reactivity of the gap-RPA/CRISPR-Cas12a assay against clinically relevant α-globin variants.

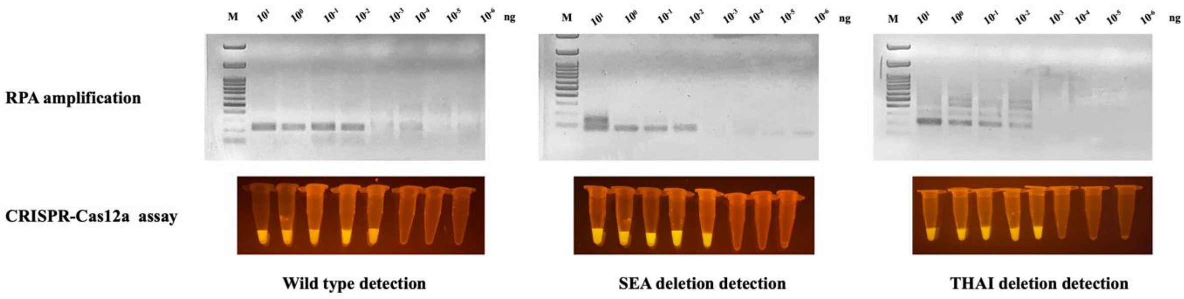

Figure S6. Analytical limit of detection of the gap-RPA/CRISPR-Cas12a assay using serially diluted genomic DNA.

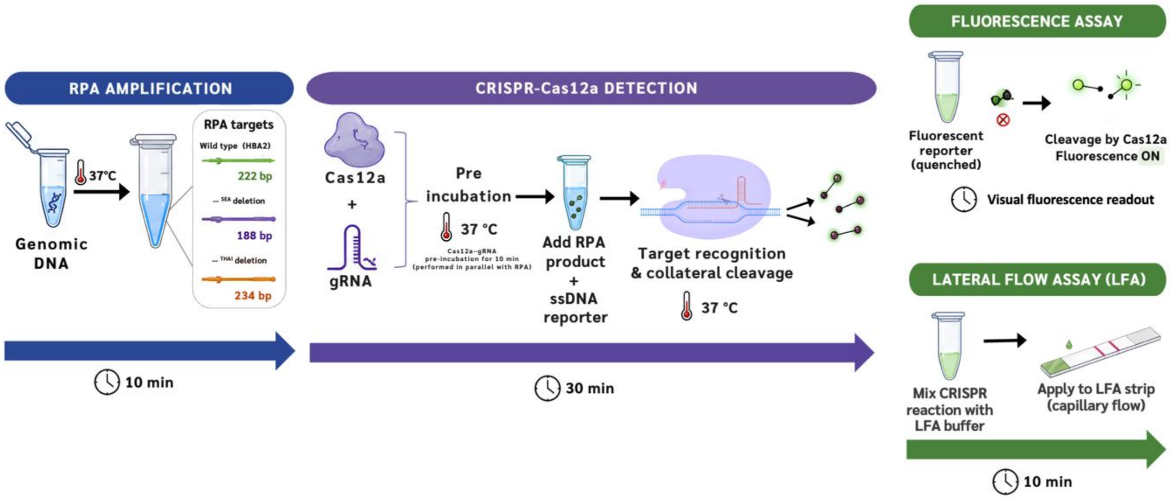

Figure S7. Schematic workflow and assay timing of the gap-RPA/CRISPR-Cas12a.
